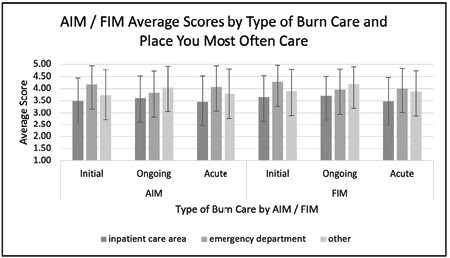# 4 Using Implementation Science to Understand Barriers to Telemedicine Use for Burn Care During a Crisis

**DOI:** 10.1093/jbcr/irae036.004

**Published:** 2024-04-17

**Authors:** Amanda Bettencourt, Jeffrey E Carter, Theresa M Davis, Katherine R Buhikire, Joseph Rhodes, Subhash Aryal, Gary Vercruysse, Deena Kelly-Costa, Colleen M Ryan

**Affiliations:** University of Pennsylvania School of Nursing, Philadelphia, Pennsylvania; University Medical Center (LSU Health), New Orleans, LA; Inova Health System, Woodbridge, University of Michigan School of Medicine, Ann Arbor, Michigan; Yale University, Orange, Connecticut; Massachusetts General Hospital/Shriners Children's, Boston, MA; University of Pennsylvania School of Nursing, Philadelphia, Pennsylvania; University Medical Center (LSU Health), New Orleans, LA; Inova Health System, Woodbridge, University of Michigan School of Medicine, Ann Arbor, Michigan; Yale University, Orange, Connecticut; Massachusetts General Hospital/Shriners Children's, Boston, MA; University of Pennsylvania School of Nursing, Philadelphia, Pennsylvania; University Medical Center (LSU Health), New Orleans, LA; Inova Health System, Woodbridge, University of Michigan School of Medicine, Ann Arbor, Michigan; Yale University, Orange, Connecticut; Massachusetts General Hospital/Shriners Children's, Boston, MA; University of Pennsylvania School of Nursing, Philadelphia, Pennsylvania; University Medical Center (LSU Health), New Orleans, LA; Inova Health System, Woodbridge, University of Michigan School of Medicine, Ann Arbor, Michigan; Yale University, Orange, Connecticut; Massachusetts General Hospital/Shriners Children's, Boston, MA; University of Pennsylvania School of Nursing, Philadelphia, Pennsylvania; University Medical Center (LSU Health), New Orleans, LA; Inova Health System, Woodbridge, University of Michigan School of Medicine, Ann Arbor, Michigan; Yale University, Orange, Connecticut; Massachusetts General Hospital/Shriners Children's, Boston, MA; University of Pennsylvania School of Nursing, Philadelphia, Pennsylvania; University Medical Center (LSU Health), New Orleans, LA; Inova Health System, Woodbridge, University of Michigan School of Medicine, Ann Arbor, Michigan; Yale University, Orange, Connecticut; Massachusetts General Hospital/Shriners Children's, Boston, MA; University of Pennsylvania School of Nursing, Philadelphia, Pennsylvania; University Medical Center (LSU Health), New Orleans, LA; Inova Health System, Woodbridge, University of Michigan School of Medicine, Ann Arbor, Michigan; Yale University, Orange, Connecticut; Massachusetts General Hospital/Shriners Children's, Boston, MA; University of Pennsylvania School of Nursing, Philadelphia, Pennsylvania; University Medical Center (LSU Health), New Orleans, LA; Inova Health System, Woodbridge, University of Michigan School of Medicine, Ann Arbor, Michigan; Yale University, Orange, Connecticut; Massachusetts General Hospital/Shriners Children's, Boston, MA; University of Pennsylvania School of Nursing, Philadelphia, Pennsylvania; University Medical Center (LSU Health), New Orleans, LA; Inova Health System, Woodbridge, University of Michigan School of Medicine, Ann Arbor, Michigan; Yale University, Orange, Connecticut; Massachusetts General Hospital/Shriners Children's, Boston, MA

## Abstract

**Introduction:**

During a crisis, hospitals will struggle to meet the care needs of burn patients. Very few clinicians (1% of MDs and RNs) and few hospitals (2%) have burn care expertise. Due to these capacity limitations, patients with burns as large as 40% TBSA will likely have to remain outside of burn centers for days to weeks before reaching definitive care. Telemedicine technology (TT) is an effective way to connect a caregiver in any location to an expert burn clinician, however it remains underused for unknown reasons. Implementation science seeks to uncover the factors affecting the use of innovations like telemedicine with the goal of increasing uptake.

**Methods:**

We administered a questionnaire to assess burn (BC) and emergency department (ED) clinician perceptions of the feasibility, acceptability, and intention to use TT across a network of 24 hospitals representing 4 of the 6 current ABA disaster response regions. We asked clinicians to respond to both crisis care (initial and ongoing) and routine acute. A Likert scale in the previously-validated acceptability of intervention measure (AIM), feasibility of intervention measure (FIM), and technology acceptance model tool (STAT) were used. Descriptive statistics were generated using SAS software. We received a total of 389 clinician responses, reflecting a 60% response rate from BC and a 7% response rate from ED staff.

**Results:**

On average, clinician ratings of the acceptability of using TT for routine burn care were higher in the ED clinicians than those working in BCs (4.07, SD=0.87 vs 3.45, SD=1), and this trend was consistent for initial and ongoing care during a crisis (4.17; SD=0.77 vs 3.48; SD=0.96 and 3.83; SD=0.89 vs 3.71; SD=0.8, respectively). We observed similar trends by practice location in feasibility and intention to use during a crisis across the sample. There were consistently positive ratings in the STAT tool domains of intention, attitude, and perceived usefulness across all clinicians regardless of practice location, however ease of use and understanding the process were consistently rated lower.

**Conclusions:**

BC and ED clinicians must work together to use telemedicine technology to treat and triage burn-injured patients during a crisis, however clinicians in burn centers currently rate its acceptability and feasibility, and their intention to use it lower than ED clinicians. Our results reveal that to improve the uptake of TT under usual care and during a crisis, attention to improving its ease of use and clinician understanding of the process is key

**Applicability of Research to Practice:**

Results suggest that BCs need to partner closely with EDs so processes for the use of telemedicine can be established and supported. To realize the benefit of telemedicine technology in burn care, an implementation intervention that improves clinician perceptions of acceptability and feasibility is warranted, and that intervention should be tailored to fit the context where the clinicians practice.